# The importance of selection in the evolution of blindness in cavefish

**DOI:** 10.1186/s12862-017-0876-4

**Published:** 2017-02-07

**Authors:** Reed A. Cartwright, Rachel S. Schwartz, Alexandra L. Merry, Megan M. Howell

**Affiliations:** 1 The Biodesign Institute, Arizona State University, Tempe, AZ USA; 2 School of Life Sciences, Arizona State University, Tempe, AZ USA; 3 Barrett, The Honors College Arizona State University, Tempe, 85287 AZ USA

**Keywords:** Migration-selection balance, Population genetics, Models/simulations, Mutations

## Abstract

**Background:**

Blindness has evolved repeatedly in cave-dwelling organisms, and many hypotheses have been proposed to explain this observation, including both accumulation of neutral loss-of-function mutations and adaptation to darkness. Investigating the loss of sight in cave dwellers presents an opportunity to understand the operation of fundamental evolutionary processes, including drift, selection, mutation, and migration.

**Results:**

Here we model the evolution of blindness in caves. This model captures the interaction of three forces: (1) selection favoring alleles causing blindness, (2) immigration of sightedness alleles from a surface population, and (3) mutations creating blindness alleles. We investigated the dynamics of this model and determined selection-strength thresholds that result in blindness evolving in caves despite immigration of sightedness alleles from the surface. We estimate that the selection coefficient for blindness would need to be at least 0.005 (and maybe as high as 0.5) for blindness to evolve in the model cave-organism, *Astyanax mexicanus*.

**Conclusions:**

Our results indicate that strong selection is required for the evolution of blindness in cave-dwelling organisms, which is consistent with recent work suggesting a high metabolic cost of eye development.

**Electronic supplementary material:**

The online version of this article (doi:10.1186/s12862-017-0876-4) contains supplementary material, which is available to authorized users.

## Background

Blindness has evolved repeatedly across taxa in caves, creating nearly a thousand cave-dwelling species and many more sub-populations [1–4]. Surprisingly, many populations of blind individuals experience some level of immigration, which would be expected to prevent the fixation of blindness in a newly established population [3–5]. Thus, blind cave-dwelling populations of typically sighted species pose an interesting challenge to our understanding of evolutionary biology. Namely, how does significant population differentiation evolve despite homogenizing immigration?

Several hypotheses have been put forward to explain the evolution of blindness in cave-dwelling species. Darwin suggested that eyes would be lost by “disuse” [6]. We now consider this hypothesis the “neutral-mutation hypothesis” — random mutations can accumulate in genes or regulatory regions related to sight when, as in caves, there is no purifying selection to eliminate them. However, the accumulation of mutations causing blindness due to mutation pressure would take a long time to result in fixation of blindness in populations on its own [7]. Thus, genetic drift has been proposed to accelerate the evolution of blindness due to mutation pressure [8–10].

Relaxing selection that maintains the eye, however, also allows for other agents of selection to act on this trait [11]. The “adaptation hypothesis” suggests that there is a cost to an eye; thus, individuals without eyes have greater fitness when eyes are not helpful, resulting in the eventual elimination of seeing individuals. This cost may either come from the energy required to develop a complex structure or due to the vulnerability of the eye [7, 12–16]. Alternatively, blindness may evolve not due to direct selection but due to selection for another beneficial trait which results in reduced eye development through pleiotropy [13].

Much of the work on the evolution of blindness has focused on cavefishes. The Mexican tetra (*Astyanax mexicanus*), which inhabits surface waters and cave systems in Mexico, is the most studied species of cave-dwelling fish. Surface and cave forms of this species are distinct, but can hybridize. The neutral-mutation hypothesis appears to be supported in this cavefish by the observation of a high number of substitutions in putative eye genes [17–19]. In addition, no differences have been found in the survival rate for blind and seeing forms between dark and light conditions [20].

The adaptation hypothesis has also been supported by work in this species. Analysis of quantitative trait loci suggests that selection has acted directly to reduce eyes in the cave populations [14]. Due to a high metabolic cost of developing and maintaining eye tissue [21], blind fish may have been favored by low resource availability in the dark caves. However, several lines of evidence do not support blindness evolving for energy conservation [13]. An alternative hypothesis is that selection for improved feeding leads to pleiotropic eye loss without direct selection for blindness. Increased Hedgehog signaling affects feeding structures, allowing for better foraging, but also causes the degeneration of eye tissue [13, 22, 23].

While it is clear that direct or indirect selection can lead to blindness despite immigration, the level of selection required to induce blindness in cave populations has not been quantified. Here, we model the effects of migration, selection, and mutation to determine the conditions required for the evolution of blindness. This model allows us to explore migration-selection-mutation balance. Previous theoretical work has explored this balance generally [24–31]. However, understanding the the evolution of cavefish requires application to this system specifically. For example, in this system, unlike many examples of local adaptation related to the continent-island model, the two populations are nearly fixed for opposite conditions of a trait. Here, we address cavefish evolution specifically by allowing for new mutations and multiple loci potentially related to blindness. We also focus on population parameters specific to cavefish. We find that the amount of selection required to oppose the force of immigration is high, but consistent with previous work on metabolic costs in novel environments and selection in other species. Interestingly, drift only impacts blindness in the cave population in a limited range of combinations of selection, dominance, and migration.

## Methods and results

### Assumptions

We consider two populations: surface-dwelling and cave-dwelling. We are interested in determining when the cave population will evolve blindness, i.e. become mostly comprised of blind individuals, as has occurred in numerous natural systems. We first assume that the surface and cave populations do not experience drift (i.e. populations are of infinite size). Additionally, immigration from the surface population into the cave affects the allele frequency in the cave population, but emigration from the cave to the surface does not affect the surface population, as we assume that the surface population is significantly larger than the cave population. Generations are discrete and non-overlapping, and mating is random. We track a single biallelic locus, where *B* is the sightedness allele and where *b* is the blindness allele.

The frequency of *b* is denoted by *Q*∈ [ 0,1] in the surface population and *q*∈ [ 0,1] in the cave population. On the surface, we assume that blindness is strongly selected against, and *Q* is dictated by mutation-selection balance. These and all subsequent variables are described in Table 1.

**Table 1:** Terms and variables

*B*	An allele that causes sightedness
*b*	An allele that causes blindness
*q*	Allele frequency of *b* in the cave population
*Q*	Allele frequency of *b* in the surface population
*q* ^′^	Frequency of *b* in the cave population in the next generation
*Δ* *q*	Change in allele frequency in a generation
*s*	Fitness advantage of *b* in the cave population
*h*	Dominance level of *b* in heterozygotes
*m*	Rate of immigration from the surface population to the cave population
*u*	Mutation rate of *B* to *b*
*k*	Number of additive diploid loci
*N*	The size of the population in the cave

### Calculating the frequency of the blindness allele

Within the cave, the life cycle is as follows. (1) Embryos become juveniles and experience constant, directional selection with relative fitnesses of *w*_*bb*_=1+*s, w*_*Bb*_=1+*hs*, and *w*_*BB*_=1, where *s*≥0 and *h*∈ [ 0,1]. (2) Juveniles migrate into and out of the cave such that a fraction *m* of adults come from the surface and 1−*m* from the cave, where 0≤*m*≤1. (3) Adults generate gametes with one-way mutation, where 0≤*u*≤1 is the probability that a functional *B* allele becomes a non-functional *b* allele. (4) Gametes unite randomly to produce embryos. Given this life cycle, we calculate the allele frequency of the daughter generation (*q* ^′^) via standard equations:

1a $$\begin{array}{*{20}l} & q_{j}= \!\frac{(1+s)q^{2}+ (1+hs)q (1-q)}{(1+s)q^{2}+2(1+hs)q (1-q)+(1-q)^{2}} \quad \text{selection} \end{array} $$

1b $$\begin{array}{*{20}l} & q_{a} = q_{j}(1-m) + Q m \qquad\qquad\qquad \text{immigration} \end{array} $$

1c $$\begin{array}{*{20}l} & q^{\prime} = q_{a} + (1-q_{a}) u \qquad\qquad\qquad \;\;\; \text{mutation} \end{array} $$

#### Analysis of the change in allele frequency

The change in allele frequency in one generation is *Δ* *q*=*q* ^′^−*q*. The first derivative of the dynamics is informative about the behavior of the model under the influence of the different parameters. Selection and mutation are directional forces, and increasing *s* or *u* increases *Δ* *q* for 0≤*q*≤1 (i.e. derivatives are non-negative). Increasing *h* causes selection to be more effective at low *q*, as rare *b* alleles are exposed to selection, but less effective at high *q*, as rare *B* alleles are sheltered from selection; increasing *h* increases *Δ* *q* if $0 < q < {\left (1+\sqrt {1+s}\right)}^{-1}$ and decreases it if ${\left (1+\sqrt {1+s}\right)}^{-1} < q < 1$ (i.e. the derivative is positive below this threshold and negative above it). Migration harmonizes the allele frequency in the cave population towards the surface population allele frequency. Thus increasing *m* increases *Δ* *q* for low *q* and decreases *Δ* *q* for high *q* (i.e. the derivative is positive only when 0≤*q*<*q*_*z*_(*h,s,Q*)≤*Q*, where *q*_*z*_ is a function describing a threshold). However, increasing *Q* increases *Δ* *q* for 0≤*q*≤1 (i.e. the derivative is non-negative).

### Identifying equilibrium allele frequencies

**Fig. 1:**
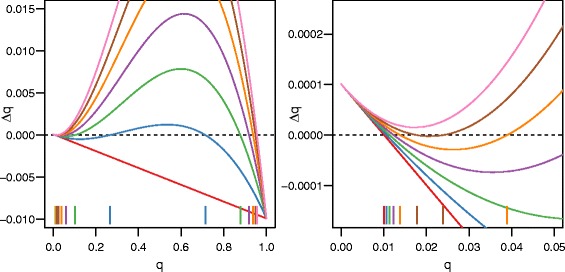
As selection increases, the evolutionary dynamics of the cave population changes. When *s* is low (*red line*; *s*=0), there is only one equilibrium: near 0. As *s* increases (*blue–brown lines*, *s*=0.05,0.1,0.15,0.2, and 0.25) the local maximum (upper hump) increases and crosses the x-axis, producing three equilibria. When *s* gets high enough (*pink line*; *s*=0.3), the local minimum (lower valley) also crosses the x-axis, resulting in one equilibrium again. The location of the equilibria are marked using vertical lines at the bottom of the chart. For all curves *m*=0.01, *h*=0, *u*=10^−6^, and *Q*=0.01. The figure on the right is an enlarged view of a small part of the figure on the left

The model we have developed is an example of migration-selection balance [26–28], extended to also include mutation. An equilibrium exists for this model when *Δ* *q*=0. For small *s*, there is only one equilibrium, and it is near 0. For large *s*, there is only one equilibrium, and it is near 1. Three equilibria will only exist for moderate levels of selection (Fig. 1). If *s*=*m*=*u*=0, all forces of evolution are eliminated and *Δ* *q*=0 for 0≤*q*≤1. A lower bound for any valid equilibrium is $\frac {mQ(1-u)+u}{m(1-u)+u}$ (Proposition 1). An upper bound for any equilibrium is 1−*m*(1−*u*)(1−*Q*) (Proposition 2). Furthermore, it is important to note that

2 $$ Q \le \frac{mQ(1-u)+u}{m(1-u)+u} \implies Q \le \hat{q}   $$

indicating that the equilibrium frequency in the cave population will be greater than or equal to the allele frequency in the surface population. Intuitively, this result is obvious as positive selection and one-way mutation only add to the frequency of the blindness allele in the cave.

Assuming *s*>0, the solution to *Δ* *q*=0 are the roots of the following cubic polynomial

3 $$ g(q) = A q^{3} + Bq^{2} + Cq + D = 0   $$

where

$$ \begin{aligned} A &= -s(1-2 h)\\ B &= s(1\,-\,m(1\,-\,u)(1\,-\,Q)\,-\,h(3\,+\,u\,-\,m(1-u)(1-2Q)))\\ C &= -(m (1-u)+u)+sh(1+u - m(1-u)(1-2Q))\\ D &= Q m (1-u)+u \end{aligned} $$

There are three possible roots of this equation, corresponding to three possible equilibria. Depending on the parameter values, Eq. 3 may have three real roots or one real root and two imaginary roots. While the values of the roots of this polynomial can be expressed analytically, these equations are too complex to be helpful for understanding the system. For simplicity, we will let $\hat {q}$ represent any possible equilibrium, and $\hat {q}_{a} \le \hat {q}_{b} \le \hat {q}_{c}$, stand for the roots of Eq. 3.

#### Protected polymorphism

Rather than tackling the equilibria directly, we first demonstrate that the cave population has a protected polymorphism. A protected polymorphism exists if the allele frequency moves away from both fixation and extinction, i.e. *Δ* *q*>0 when *q*=0 and *Δ* *q*<0 when *q*=1. For *q*=0, *Δ* *q*=*Q m*(1−*u*)+*u* and *q*=0 will be an equilibrium only if *Qm*=0 and *u*=0; otherwise *Δ* *q*>0 at *q*=0. For *q*=1,*Δ* *q*=−*m*(1−*Q*)(1−*u*) and *q*=1 will be an equilibrium if *m*=0, *Q*=1, or *u*=1; otherwise *Δ* *q*<0. Thus a protected polymorphism always exists except at the edge cases *Qm*=*u*=0,*m*=0,*u*=1, and *Q*=1. In biological terms, the cave population will be polymorphic despite directional selection for *b* if there is some immigration from the surface population and the surface population is polymorphic.

#### Validity of equilibria

An equilibrium is only valid in our model if it is real and between [ 0,1]; otherwise, it is not biologically interpretable in this system. Because there is a protected polymorphism, there will be either 1 valid, stable equilibrium, or 3 valid equilibria in a stable-unstable-stable configuration, depending on the parameter values. While we have not exhaustively determined the parameter ranges under which there will be only one valid equilibrium, we have determined that if *h*≥1/3 or if *h*<1/3 and $s h > \frac {m(1-u)+u}{1 + u - m (1-u)(1-2Q)}$, there will be only one valid equilibrium (Proposition 3).

We can also estimate the amount of selection required such that *g*(*q*)=0 (Eq. 3):

4 $$  \begin{array}{l} s_{q}(m,h,u,Q) \\ \,\,\,=\frac{m (1-u) (q-Q)-(1-q) u} {q \left(q-q\left(q+m (1-Q) (1-u)\right) -h (1-q) \left(m (1-2Q) (1-u)-(1-2 q)-u\right)+q\right)} \end{array}  $$

This equation is not valid for all *m*∈ [ 0,1]. If the migration rate is low, $m < \frac {(1-q)u}{(q-Q)(1-u)}$, no level of selection will make *q* an equilibrium, as all equilibria will be greater than *q*. Similarly, if the migration rate is high,

$$m > \frac{(1-q) \left(h (1-2 q+u)+q\right)}{(1-u) \left(h (1-q) (1-2 Q)+q (1-Q)\right)} $$

no level of selection will make *q* an equilibrium, as all equilibria will be less than *q*.

#### Dynamics and the evolution of blindness

The dynamics of the evolution of the cave population depend on the parameter values and the starting allele frequency, *q*_0_. — Our model is likely well behaved, e.g. no limit cycles or chaotic behavior, even though we provide no formal proof of this. — If there is one equilibrium, then the frequency of *b* will evolve monotonically towards it, i.e. $q_{t} \to \hat {q}$ as *t*→*∞*. If there are three equilibria, then the frequency of *b* will evolve monotonically to $\hat {q}_{a}$ if $q_{0} < \hat {q}_{b}$ and to $\hat {q}_{c}$ if $q_{0} > \hat {q}_{b}$.

When the cave population is founded, its initial allele frequency will likely match the equilibrium frequency on the surface (*q*_0_=*Q*). Because $Q < \hat {q}$ (Eq. 2), the allele frequency in the cave population will increase due to selection until it reaches the lowest equilibrium, i.e. *q*_*∞*_= inf{*q*:0≤*q*≤1 and *Δ* *q*=0}. Whether blindness evolves in the cave population depends on whether *q*_*∞*_≥*q* ^∗^, where *q* ^∗^ is a researcher-chosen threshold for determining that the cave population is a “blind” population. For example, *q* ^∗^=0.5 would specify that the blindness allele is the majority allele, and *q* ^∗^=0.99 would determine that the blindness allele is approximately fixed. We can also focus on phenotypes, and let *a*=*q* ^2^+2*q*(1−*q*)*h* measure the average blind phenotype in the cave population; then

$$a_{\infty} \ge a^{*} \implies q_{\infty} \ge \frac{\sqrt{a^{*}(1-2h)+h^{2}}-h}{1-2 h} $$

We define *s* ^∗^ as the minimum level of selection required for cave population to become blind, given the other parameters, i.e.

$$s^{*} = \inf \{ s : s > 0\ \text{and}\ q_{\infty} \ge q^{*} \gg Q\} $$

**Fig. 2:**
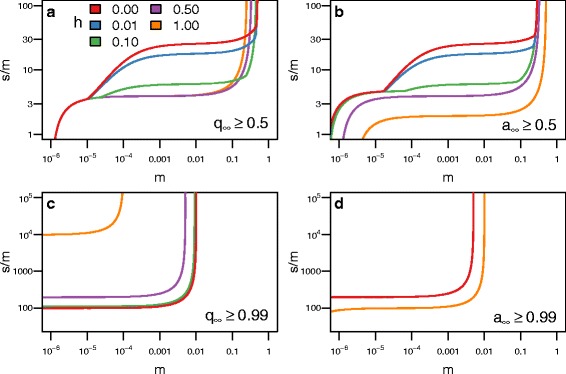
The level of dominance of the blindness allele (*h*) affects the level of selection (*s*) required to produce blind populations. Each line represents how strong selection must be relative to migration (*m*) for blindness to evolve in the cave population for a given level of dominance (*s* ^∗^/*m*), where *s* ^∗^ is the minimum level of selection required for the cave population to become blind. Regions above the curves produce populations that are blind and regions below do not. Each panel contains a different condition for defining whether the cave population is blind. **a** For the blind allele to become the majority allele requires stronger selection when blindness is recessive (*h*=0) compared to when the allele for blindness is dominant. **b** For the blind phenotype to become the majority phenotype requires stronger selection when blindness is recessive compared to when the allele for blindness is dominant. **c** For the blind allele to become fixed requires stronger selection when the allele for blindness is dominant compared to when it is recessive. **d** For the blind phenotype to become fixed requires stronger selection when blindness is recessive. The curves were calculated analytically with *u*=10^−6^ and *Q*=0.01

**Fig. 3:**
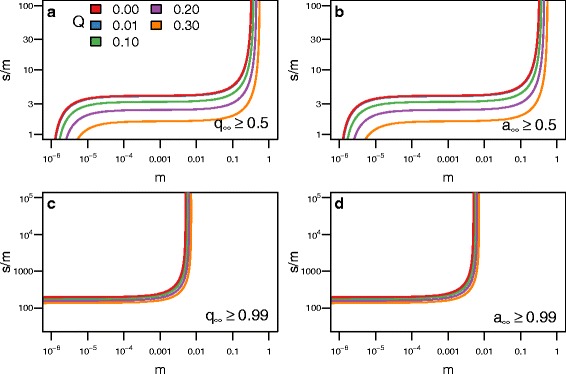
The frequency of the blindness allele (*Q*) in the surface population affects the level of selection (*s*) required to produce blind populations. The format of this figure follows Fig. 2: **a** shows when the blind allele becomes the majority allele; **b** shows when the blind phenotype becomes the majority phenotype; **c** shows when the blind allele becomes fixed; **d** shows when the blind phenotype becomes fixed. As *Q* increases, the amount of selection required to evolve blindness in the cave population decreases. A surface population with a high *Q* can be considered “pre-adapted” to the cave. The curves were calculated analytically with *u*=10^−6^ and *h*=0.5

**Fig. 4:**
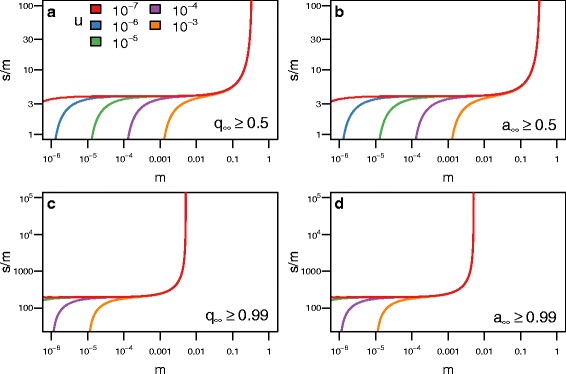
The mutation rate of the blindness allele (*u*) affects the level of selection (*s*) required to produce blind populations. The format of this figure follows Fig. 2. As *u* increases, the amount of selection required to evolve blindness in the cave population decreases for small *m*, and blindness will evolve regardless of selection. The curves were calculated analytically with *Q*=0.01 and *h*=0.5

For simplicity, we will only consider values of *q* ^∗^ much higher than the surface allele frequency. If there is one equilibrium, $\phantom {\dot {i}\!}s^{*} = s_{q^{*}}({m,h,u,Q})$; however, if there are three equilibria, *q*_*t*_ will evolve to the lower equilibrium and *q*_*∞*_≈*Q*≠*q* ^∗^ (typically). Thus selection needs to be strong enough such that there is only one equilibrium; therefore,

$$\begin{aligned} s^{*} \approx \inf &\left\{s : s > 0\ \text{and}\ s \ge s_{q^{*}}(m,h,u,Q)\right.\\ &\quad\text{and}\ \left.\Delta(s,m,h,u,Q) < 0 \right\} \end{aligned} $$

where *Δ*(*s,m,h,u,Q*) is the discriminant of Eq. 3. Figures 2, 3, and 4 plot analytical solutions for *s* ^∗^ based on different thresholds. When *m*≫*u*, the ratio *s* ^∗^/*m* is roughly constant such that if *q*_*∞*_≥*q* ^∗^ then

5 $$  {\begin{aligned} \frac{s^{*}}{m} \ge \max &\left\{ \frac{q^{*}-Q}{q^{*}(1-q^{*})\left(q^{*}+h(1-2 q^{*})\right)},\ \frac{1-6Q}{h}\right.\\ &\;\left.+\frac{2 Q-2 \sqrt{Q^{2}+ h Q \left(1-3 h (1-3 Q)-6 Q\right) }}{h^{2}} \right\} \end{aligned}}  $$

See Appendix for derivation.

#### The neutral-mutation hypothesis

If blindness evolves neutrally in the cave population (*s*=0), the equilibrium allele frequency will be governed by mutation-migration balance, i.e. $\hat {q} = \frac {mQ(1-u)+u}{m(1-u)+u}$. Similar to *s* ^∗^, we can define a critical value *m* ^∗^, such that if *m*<*m* ^∗^, the cave population will evolve blindness.

$$m^{*} = \sup \{ m : q_{\infty} \ge q^{*} \gg Q\} = \frac{(1-q^{*})u}{(q^{*}-Q)(1-u)} $$

Clearly, if *u*=0, the cave population will not evolve blindness without the influence of selection (or genetic drift). However, a completely isolated cave (*m*=0) will evolve blindness if there is mutation (*u*>0). As the migration rate increases, the equilibrium allele frequency decreases such that if *m*>*m* ^∗^, the cave population will not evolve blindness. Similarly, increasing the mutation rate increases *m* ^∗^, allowing blindness to evolve for higher immigration rates and demonstrating the importance of mutation to the evolution of blindness when selection is weak.

### Recessive blindness

If blindness is recessive (*h*=0), we can evaluate the dynamics of three equilibria in more detail. First, we will simplify our model by assuming that *u*≪1 such that 1−*u*≈1 and

6 $$ \Delta q \propto s q^{2}\left[1- q - m(1-Q)\right] + \left[Q m +u - q \left(m +u\right)\right]  $$

#### Weak-selection approximation

If selection is weak, then an equilibrium exists near *q*=*Q*. We use a second-order Taylor series at *q*=0 to determine the upper bound on *s* for the presence of three equilibria (i.e. when selection is so strong that an equilibrium near *Q* does not exist). The second-order series allows us to determine the lower two equilibrium points, although this approximation is inaccurate as q increases. This approximation gives us

7 $$ \Delta q \approx s(1-m)q^{2} - (m+u)q + mQ + u   $$

after assuming that 1−*Q*≈1. This equation has two roots, which are the lowest two of three total equilibria,

$$\begin{array}{*{20}l} \hat{q}_{a,1} &= \frac{m+u-\sqrt{(m+u)^{2}-4 s (1-m)(mQ+u)}}{2 s (1-m)}\\ \hat{q}_{b,1} &= \frac{m+u+\sqrt{(m+u)^{2}-4 s (1-m)(mQ+u)}}{2 s (1-m)} \end{array} $$

These two roots exist only if

8 $$ \begin{aligned} &0 < \sqrt{(m+u)^{2}-4 s (1-m)(mQ+u)} \implies\\ &s < \frac{(m+u)^{2}}{4(1-m)(mQ+u)} \end{aligned}   $$

which provides us with an estimate of the upper bound on *s* for the presence of three equilibria.

The derivative of Eq. 7 is $\frac {\text {d}\Delta {q}}{\text {d}q}\left (q\right) = 2 s (1-m) q - (m+u)$, and an equilibrium will be stable if $ -2 < \frac {\text {d}\Delta {q}}{\text {d}q}\left (\hat {q}\right) < 0 $. From this, it can be easily shown that $\hat {q}_{a,1}$ is stable and that $\hat {q}_{b,1}$ is unstable.

#### Strong-selection approximation

In order to determine the lower bound on *s* for the presence of three equilibria, we assume that selection is strong enough such that *u*/*s*≈0 and *Q*/*s*≈0. Therefore,

9 $$ \Delta q \propto -q\left[q^{2} - \left[1-m(1-Q)\right]q + m/s\right]   $$

and the equilibria can be described as

$$ \begin{aligned} \hat{q}_{a,2} &= 0\\ \hat{q}_{b,2} &= \frac{1}{2}\left(1-m(1-Q)-\sqrt{\left[1-m(1-Q)\right]^{2}-\frac{4m}{s}}\right)\\ \hat{q}_{c,2} &= \frac{1}{2}\left(1-m(1-Q)+\sqrt{\left[1-m(1-Q)\right]^{2}-\frac{4m}{s}}\right) \end{aligned} $$

The latter two equilibria will exist only if

$$s > \frac{4m}{\left[1-m(1-Q)\right]^{2}} $$

which provides us an estimate of the lower bound for the presence of three equilibria.

The derivative of Eq. 9 is $\frac {\text {d}\Delta {q}}{\text {d}q}\left (q\right) = -3 q^{2} + 2 [1-m$ (1−*Q*)]*q*−*m*/*s*, and it can be easily shown that $\hat {q}_{b,2}$ is unstable and $\hat {q}_{c,2}$ is stable.

#### Validity of approximations

**Fig. 5:**
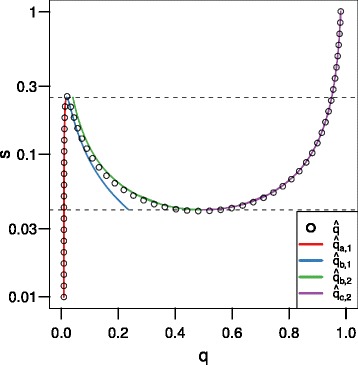
Our recessive-blindness equilibria approximations are accurate. The approximations developed in this paper (solid lines) are a good fit for calculated values of selection (*s*) that result in equilibrium for a given frequency of the blindness allele (*q*; circles) using Eq. 3. The dashed lines are our approximate bounds for the existence of three equilibria (i.e. for small and large values of *s*, there is one equilibrium; for intermediate values of *s* there are three possible equilibria). Other parameters are *m*=0.01, *u*=10^−6^, and *Q*=0.01

By substituting $\hat {q}_{a,1}$ and $\hat {q}_{b,1}$ back into Eq. 6, we obtain $\Delta q = - s \hat {q}^{2}\left (\hat {q}-Qm\right)$. Thus, *Δ* *q*≤0, which indicates that $\hat {q}_{a,1}$ overestimates $\hat {q}_{a}$ and that $\hat {q}_{b,1}$ underestimates $\hat {q}_{b}$. By substituting $\hat {q}_{b,2}$ and $\hat {q}_{c,2}$ back into Eq. 6, we find that $\Delta q = Qm+u(1-\hat {q})$. Thus *Δ* *q*≥0, which indicates that $\hat {q}_{b,2}$ overestimates $\hat {q}_{b}$ and that $\hat {q}_{c,2}$ underestimates $\hat {q}_{c}$. However, the error in our approximations is slight (Fig. 5).

#### Dynamics

Based on these approximations, the dynamics of the recessive-blindness system can be summarized as follows. First, there are three possible equilibria: $\hat {q}_{a} \approx \hat {q}_{a,1}$, $\hat {q}_{b} \in \left [ \hat {q}_{b,1},\hat {q}_{b,2} \right ]$, and $\hat {q}_{c} \approx \hat {q}_{c,2}$. Second, there are four possible equilibria configurations: 1, 2a, 2b, and 2c.

Case 1, $\frac {(m+u)^{2}}{4(1-m)(mQ+u)} < \frac {4m}{\left [1-m(1-Q)\right ]^{2}}$: only one equilibrium exists, and it is stable. The population will always evolve towards it.

Case 2, $\frac {4m}{\left [1-m(1-Q)\right ]^{2}} < \frac {(m+u)^{2}}{4(1-m)(mQ+u)}$: depending on the strength of *s*, this case may have one of three possible configurations:

Case 2a, $0 \le s < \frac {4m}{\left [1-m(1-Q)\right ]^{2}}$: Only one equilibrium exists, $\hat {q}_{a}$, and it is stable. The population will always evolve towards it.

Case 2b, $\frac {4m}{\left [1-m(1-Q)\right ]^{2}} < s < \frac {(m+u)^{2}}{4(1-m)(mQ+u)}$: All three equilibria exist; $\hat {q}_{a}$ and $\hat {q}_{c}$ are stable, while $\hat {q}_{b}$ is unstable. If the population starts below $\hat {q}_{b}$, it will evolve towards $\hat {q}_{a}$. If it starts above $\hat {q}_{b}$, it will evolve towards $\hat {q}_{c}$.

Case 2c, $\frac {(m+u)^{2}}{4(1-m)(mQ+u)} < s$: only one equilibrium, $\hat {q}_{c}$, exists, and it is stable. The population will always evolve towards it.

Furthermore if *q*_0_=*Q*, the selection-threshold for blindness to be established in the cave population is

10 $$ {\begin{aligned} s^{*} \approx \max \left\{ \frac{m(q^{*}-Q)-u(1-q^{*})}{q^{*2}\left(1-q^{*}-m(1-Q)\right)},\ \frac{(m+u)^{2}}{4(1-m)(mQ+u)} \right\} \end{aligned}}  $$

where *q* ^∗^ is the allele-frequency threshold.

### Additive blindness and multiple alleles

Next we investigate a model where blindness is due to many additive (*h*=0.5) loci of small effect. This model is motivated by the identification of 12 additive loci corresponding to the difference in eye phenotypes between cave and surface populations of *A. mexicanus* [14]. First, we will make the following assumptions: (1) there are *k* unlinked loci with two alleles (for sightedness and blindness), (2) in the cave population the fitness of an individual is $1 + s \frac {x}{2k}$, where *x* is the number of blindness alleles the individual carries, and (3) *m*, *u*, *Q*, and *q*_0_ are identical at each locus.

Because the forces of evolution are equivalent at every locus, they will evolve identically, and the change in allele frequency due to selection is

11 $$ q_{j} = q \frac{1+q s + (1-q) \frac{s}{2k}}{1+q s}  $$

See the appendix for a derivation. Next, we will simplify our model by assuming that *u*≪1 such that 1−2*k u*≈1 and

12 $$ \begin{aligned} \Delta q \propto &-s(1 - (2k -1)m) q^{2} + (s (1 - m (1 - 2 k Q))\\ &- 2 k (m +u)) q +2 k (Q m + u) \end{aligned}  $$

This has a single, stable, valid equilibrium:

$$\begin{aligned} \hat{q} = \frac{s (1 - m (1 - 2 k Q))- 2 k (m+u) + \sqrt{(2 k (m (Q s-1)-u)-m s+s)^{2}+8 k s ((2 k-1) m+1) (m Q+u)}}{2 s ((2 k-1) m+1)} \end{aligned} $$

which decreases a *k* increases. Furthermore, if *m*≫*u*

$$\frac{s^{*}}{m} \ge \frac{2 k (q^{*}-Q)}{q^{*}(1-q^{*})} $$

In summary, the frequency of blindness alleles will increase in the cave population until they reach equilibrium, and they will be majority alleles if *s*≥*s* ^∗^>4*k m*.

### Finite-population simulations

#### Constant migration

Cavefish live in small populations and strong levels of drift may play a significant role in the evolution of blindness in cave species. To investigate the impact of drift on our recessive-blindness model, we simulated diploid populations of size *N*=1000 (based on population estimates by [3]) by modifying our life cycle (Eq. 1) to include a finite population:

13a $$\begin{array}{*{20}l} q_{j} &= \frac{(1+s)q^{2}+ q (1-q)}{(1+s)q^{2}+(1-q^{2})} & & \text{selection} \end{array} $$

13b $$\begin{array}{*{20}l} q_{m} &= q_{j}(1-m) + Q m & & \text{immigration} \end{array} $$

13c $$\begin{array}{*{20}l} q_{a} &\sim \text{Binomial}(q_{m}, 2N) / 2 N & & \text{drift} \end{array} $$

13d $$\begin{array}{*{20}l} q^{\prime} &= q_{a} + (1-q_{a}) u & & \text{mutation} \end{array} $$

Here the adult population consists of 2*N* alleles sampled with replacement from the post-immigration gene pool.

For every simulation, *u*=10^−6^ and *Q*=0.01. These values were chosen because they are believed to be reasonable estimates, and because we previously examined the impact of varying *Q* and *u* (Figs. 3 and 4). We further explain the impact of altering these choices in the discussion. We set *q*_0_=*Q*, varied *s* from 10^−6^ to 10^2^, and varied *m* from 10^−8^ to 1.

We simulated 100 replicates for each combination of parameters; simulations were conducted for 10,000 or 5,000,000 generations. For each set of parameters, we recorded the average *q* ^′^ frequency across these 100 populations at specific time points.

**Fig. 6:**
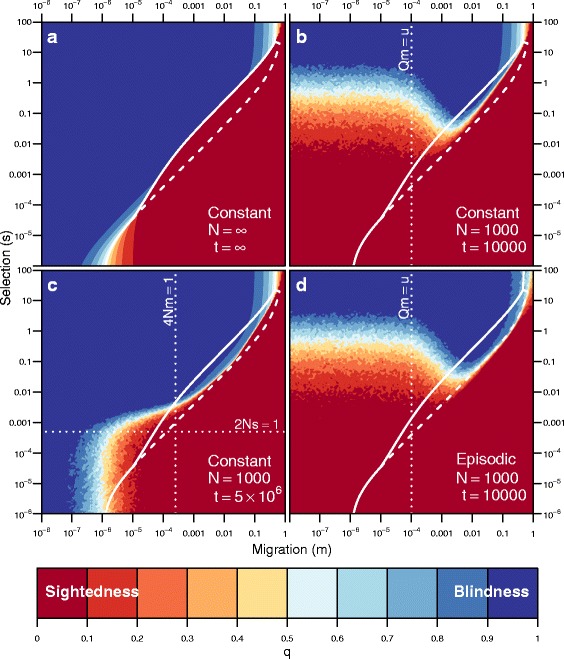
Populations evolve blindness in the face of immigration only with the help of strong selection. **a** The equilibrium frequency of the blindness allele (*q*) for an infinite population, and **b**–**d** average frequencies of the allele after *t* generations in finite populations with either constant or episodic migration. For each combination of selection (*s*) and migration (*m*) we conducted 100 replicate simulations with fixed values of the mutation rate (*u*=10^−6^), frequency of the blindness allele in the surface population (*Q*=0.01), and *q*_0_=*Q*. *Colors* correspond to the frequency of the blindness allele for a given combination of *s* and *m*, where *blue* is high frequency (blindness evolved) and *red* is low (blindness did not evolve). The *solid white line* corresponds to the degree of selection required in the infinite population (**a**) to result in *q*_*∞*_>0.5 ($s^{*}_{0.5}$). The area between the *solid* and *dashed lines* corresponds to the region where three equilibria exist. If 2*N s*≪1, drift is stronger than selection, and if 4*N m*≪1, drift is stronger than migration. If *m Q*≪*u*, mutation is the primary force introducing copies of the blindness alleles to the cave population

Our simulation results for finite populations are qualitatively similar to our analytical results for infinite populations. For high migration rates, the average allele frequency is similar to the infinite model, except that drift allows some populations that have three equilibria to evolve blindness (Fig. 6 b). However, at low migration rates (*Q m*<*u*), populations have low average frequency of *b* at 10,000 generations, unless *s*>1. As immigration decreased, these populations became dependent on *de novo* mutations to produce *b*, which is a slow process. At 5 million generations, which is close to the estimated age of cavefish populations [32], the average allele frequency is a better match to the results from the the infinite-population model (Fig. 6 c); however, it differs in two respects. (1) When selection is ineffective (2*N s*<1), the average allele frequency reflects mutation-migration balance. And (2) when migration is low (4*N m*<1), the average allele frequency shows increased variation. Thus drift is the strongest force affecting the change in allele frequencies in the bottom left of Fig. 6 c. For *N*=100 (not shown), results are qualitatively similar, but stronger selection is required to overcome the stronger effects of drift present at smaller population sizes, which most often leads to loss of the rare blindness allele.

#### Episodic migration

Because cave and surface populations may be connected intermittently due to flooding, we simulated periods of immigration followed by periods of isolation following a first-order Markov process. The probability of switching between from isolation to immigration or vice versa was 10% in each generation. Results for the intermittently connected simulations were nearly identical to previous simulations, with the exception that at high levels of migration and selection, drift was more effective in increasing average allele frequencies (Fig. 6 d).

#### Multiple loci

To determine the effects of drift with multiple loci, we implemented the following individual-based simulation:

14a $$\begin{array}{*{20}l} q_{j,i} &= \bar{w}^{-1} \sum_{a=1}^{N}{w_{a} q_{a,i}} & & \text{selection} \end{array} $$

14b $$\begin{array}{*{20}l} q_{m,i} &= q_{j,i}(1-m) + Q m & & \text{immigration} \end{array} $$

14c $$\begin{array}{*{20}l} q_{u,i} &= q_{m,i} + (1-q_{m,i}) u & & \text{mutation} \end{array} $$

14d $$\begin{array}{*{20}l} q_{a,i}^{\prime} &\sim \text{Binomial}(q_{u,i}, 2) / 2 & & \text{drift} \end{array} $$

where *q*_*a,i*_ is the frequency of blindness allele at the *i*-th locus in the *a*-th individual, *w*_*a*_ is the fitness of the *a*-th individual, and $\bar {w}$ is the average fitness. Note that we use fecundity selection in this simulation to reduce its complexity. Simulations of 1000 individuals were run for 10,000 generations with *u*=10^−6^, *Q*=0.01, and a grid of *s* and *m* values. The number of loci was *k*∈{1,2,4,6,12}. For each parameter value, 100 simulations were run and several summary statistics were calculated: the average frequency of blindness alleles, the average fitness, the average phenotype, and the average genetic load in the cave population.

**Fig. 7:**
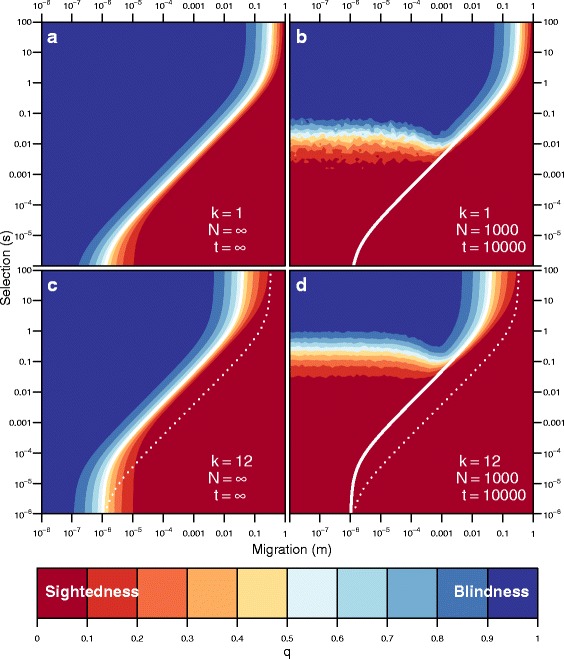
Many loci of small effect require stronger selection than a a single locus of large effect to evolve blindness in the face of immigration. **a** The equilibrium frequencies of the blindness allele for an infinite population with a single locus and **b** average frequencies of the allele after *t* generations in finite populations of size *N* (100 replicates). **c** and **d** The impact of multiple loci (*k*=12) on the evolution of blindness. *Colors* correspond to the frequency of the blindness allele (*b*) for a given combination of selection (*s*) and migration (*m*), where *blue* is high frequency (blindness evolved) and *red* is low (blindness did not evolve). The *solid white lines* correspond to $s^{*}_{0.5}$ for *k*=1 or 12 and the *dashed lines* correspond to $s^{*}_{0.5}$ for *k*=1

Our stochastic simulations agree with our deterministic results (Fig. 7 shows infinite and finite results for *k*=1 and *k*=12). As predicted by the deterministic model, increasing the number of loci increased the amount of selection required to evolve blindness in the cave population. This result is due to the fact that genes with smaller effect size show more genetic load due to migration of surface individuals into the cave. Even when migration was weak, a smaller effect size decreased the strength of selection relative to drift.

## Discussion

The evolution of blindness in caves has been hypothesized to result from relaxed selection and mutation pressure and/or positive selection for alleles that result in eye loss. However, the degree to which these factors interact and the theoretical level of selection required to induce blindness have not been quantified previously.

Here we have shown that for blindness to evolve via neutral processes, immigration must be rarer than mutation, i.e. the cave must be almost completely isolated from the surface. If the cave is not completely isolated from the surface, immigration of surface fish will make it nearly impossible for blindness to evolve in the cave population without strong selection favoring the trait. When determining whether blindness evolves neutrally or adaptively, it is also important to consider how old cave populations are. If a blind cave population is much younger than *u* ^−1^ generations, then it is likely that selection was influential in driving the rapid evolution of the cave population.

### Effects of genetic drift

Cave populations are likely to be small, and intuitively genetic drift should play a role in the evolution of blindness in the cave population. In most cases, blindness alleles are rare in the surface population, and drift is expected to lead mostly to sighted populations [20]. Drift also reduces the strength of natural selection such that 2*Ns*≫1 for adaptive processes to function (Fig. 6 c). Therefore, smaller populations require stronger levels of selection to eliminate migration load and evolve blindness. Interestingly, drift is only *essential* to the evolution of blindness in the cave population in a limited range of combinations of selection and migration for which we find three equilibria.

### Effects of dominance

The amount of selection required for blindness to evolve depends on the migration rate and the level of dominance of the blindness allele (Fig. 2). For example, if *Q*=0.01 and *h*=0, the amount of selection needs to be about 25 times the migration rate for a blind allele to become the major allele. Conversely, if *h*>1/3, the amount of selection only needs to be about three times the migration rate. The situation is reversed when we look at fixation. If *h*=0, selection needs to be about 100 times the migration rate for the frequency of the blind allele to exceed 99% in the cave population, and if *h*=1, it needs to be 10,000 times greater than the migration rate. If we focus on phenotypes instead, we see that dominant alleles need lower levels of positive selection to impact the population (Fig. 2).

### Effects of multiple loci

Increasing the number of unlinked loci underlying the blindness phenotype increases the amount of selection needed to evolve blindness. Intuitively, blindness alleles with small effect reduce migration load less effectively in the cave population. With all things being equal, a single allele of large effect would be more likely to sweep through the cave population than multiple alleles of small effect. However, if alleles of large effect tend to be recessive, they would be less likely to sweep than alleles of small effect that are additive.

### Magnitude of selection

The magnitude of selection coefficients required by our model to produce blindness given modest levels of immigration are comparable to observations in many species. Levels of selection sufficient to produce selective sweeps in wild populations range from 0.02–0.7 [33–36]. Estimated selection coefficients for drug resistance in *Plasmodium falciparum* were 0.1–0.7, leading to fixation in 20–80 generations [35, 36]. For a major advantageous allele, the average value of *s* has been estimated as 0.11 in plants and 0.13 in animals [37, 38].

The well-studied three-spine stickleback (*Gasterosteus aculeatus*) exhibits similar strong selection in a novel environment. In experiments isolating armored sticklebacks in freshwater pools, armor was lost within a few generations due to relaxed selection for defense and positive selection for the lower cost of development in unarmored fish [39]. Estimates of selection in this species have ranged from 0.13–0.16 [40].

The selection coefficient of a blindness allele is determined not only by its impact on the visual system, but also by any other pleiotropic effects, such as enhancement to feeding ability [13]. If an allele produces multiple, adaptive phenotypes, its selection coefficient is more likely to be high enough to promote local adaptation and differentiation between cave and surface populations.

### Understanding *Astyanax mexicanus*

*A. mexicanus* is the most well studied cave dwelling species. This species has inhabited caves for approximately 2–3 million years [32] or 5 million generations (generation time is 4–6 months in the lab [41]). Given this amount of time, neutral processes might explain the evolution of blindness in cave populations if they were completely isolated. However, the cave populations are not isolated from the surface populations; they receive immigrants at a rate of 10^−4^– 10^−2^ per generation [3, 42]. While no fitness difference was detected in laboratory experiments [20], there are 12 QTLs identified for eye-related phenotypes, and they show a signature of selection directly favoring regressive phenotypes in cave populations [14]. This selection may be due to eye development imposing a high metabolic cost, particularly for juveniles [21], such that individuals with regressive eye-phenotypes require less resources. However, the precise degree of this selection is unknown.

In this species, our additive model predicts that *s* would need to be about 48 times stronger than *m* for the 12 identified, cave-related alleles [14] to become the majority alleles in cave populations. For selection to be effective in cave populations (*N*_*e*_ ranges between 400 and 1400 [3, 42]) *s* would *at minimum* need to be between 0.005 and 0.5. If we required allele frequencies to reach 90%, these estimates would be five times higher! These estimates are high, but within the range of results found previously for selective sweeps (discussed above). We recognize that these estimates could be improved if data was available for surface allele frequencies, mutation rates, and dominance values.

These coefficients are high enough that laboratory experiments could have detected a difference between surface and cave forms; however they did not [20]. One possible explanation of this discrepancy is that selection for blindness is due not to survival or reproductive success but to genotype-dependent dispersal [43, 44]. Ninety years ago, Lankester [45] proposed that blindness evolves in caves because fish with eyes may be attracted to sources of light and preferentially leave caves. Emigration of fish from the caves to the surface is common in *A. mexicanus* [3, 42]. In our model, emigration of sighted individuals would act like selection, because individuals with sightedness alleles would systematically leave the cave and not contribute to the gene pool. Even a low level of preferential emigration, e.g. 2%, would provide a significant boost to local adaptation and the evolution of blindness in caves. It is quite possible that genotype-dependent dispersal combined with lower development costs promotes the elimination of sight in caves despite the immigration of sightedness alleles from the surface. Preferential emigration would also explain the fact that in *A. mexicanus* only the effects of eye QTLs were consistently regressive, and no other phenotypes were as consistent [14].

## Conclusion

We conclude that in most cases strong selection is necessary for the evolution of blind populations in caves. This result is consistent with two different observations of cavefish: (1) phototactic fish may leave caves, effectively selecting for the maintenance of mostly blind fish, and (2) the metabolic cost of eyes is very high. Additionally, the model and results presented in this paper are applicable beyond the evolution of cave populations, expanding existing migration-selection balance theory. We have developed approximations that allow us to understand the interaction of selection, migration, and mutation. Through simulation we have examined the effects of genetic drift relative to the model and determined that in some situations it can enhance the power of selection to drive local adaptation.

## Appendix

All the proofs below were validated in Mathematica (Additional file 1) [46].

### **Proposition 1**

If *m*>0 or *u*>0, $\frac {mQ(1-u)+u}{m(1-u)+u}$ is a possible equilibrium, and there is no equilibrium less than it. If *m*=*u*=0, 0 is an equilibrium.

### *Proof*

Case 1. Let *f*(*q*)=*q* ^′^−*q* represent the change in allele frequency over one generation (Eq. 1). Let $\tilde {q} = \frac {mQ(1-u)+u}{m(1-u)+u}$. If *s*=0 and *m*>0 (or *u*>0), $f(\tilde {q})=0$, and therefore $\tilde {q}$ is an equilibrium for these parameters. Furthermore, if *s*≥0, *f*(*q*)>0$\forall q \in \left [0,\tilde {q}\right)$. Therefore, there is no equilibrium lower than $\tilde {q}$.

Case 2. Let *m*=*u*=0, *f*(0)=0. □

### **Proposition 2**

1−*m*(1−*u*)(1−*Q*) is a possible equilibrium, and there is no equilibrium greater than it.

### *Proof*

Let $\tilde {q} = 1-m(1-u)(1-Q)$ and *h*=0. Since ${\lim }_{s \to \infty } f(\tilde {q}) = 0$, $\tilde {q}$ is a potential equilibrium. Furthermore, if 0≤*h*≤1 and *s*≥0, *f*(*q*)<0$\forall q \in \left (\tilde {q},1\right ]$. Therefore, there is no equilibrium higher than $\tilde {q}$. □

The derivation of a tighter upper bound can be achieved by not assuming *h*=0; however, we do not report it at this time.

### **Proposition 3**

Let *s*>0. Let *m*>0 or *u*>0. If *h*≥1/3 or if *h*<1/3 and $s h > \frac {m(1-u)+u}{1 + u - m (1-u)(1-2Q)}$, *g*(*q*) (Eq. 3) has exactly one root in [ 0,1].

### *Proof*

Let *m*>0 or *u*>0. Then *g*(1)<*g*(0) and *g*(1)≤0≤*g*(0). By the intermediate value theorem there is at least one root in [ 0,1]. Let *s*>0 and we will show that there is exactly one root for several cases.

Case 1. Let 1/2<*h*≤1. Then *g*(−*∞*)<0 and *g*(*∞*)>0. By the intermediate value theorem, *g*(0) has at least one root below 0, between 0 and 1, and above 1. Since *g*(0) is cubic, it can have at most 3 roots; therefore, there is exactly one root in [ 0,1].

Case 2. Let *h*=1/2. *g*(*q*) reduces to a quadratic equation with one root less than 0 and exactly one root in [ 0,1].

Case 3. Let 1/3≤*h*<1/2. $\frac {\text {d}^{2}{g(q)}}{\text {d}q^{2}} \le 0$, and *g*(*q*) is concave in [ 0,1]. Thus *g*(*q*) has exactly one root in [ 0,1].

Case 4. Let 0≤*h*<1/3 and $s h > \frac {m(1-u)+u}{1 + u - m (1-u)(1-2Q)}$. Then $\frac {\text {d}g(q)}{\text {d}q}\left (-\infty \right) < 0$, $\frac {\text {d}g(q)}{\text {d}q}\left (0\right) \ge 0$, $\frac {\text {d}g(q)}{\text {d}q}\left (1\right) \le 0$, and $\frac {\text {d}g(q)}{\text {d}q}\left (0\right) > \frac {\text {d}g(q)}{\text {d}q}\left (1\right)$. By the intermediate value theorem, there must be a local minimum in (−*∞*,0] and and a local maximum in [ 0,1]. Thus *g*(*q*) has exactly one root in [ 0,1]. □

***Derivation of Eq. 5***

In order to derive Eq. 5 we first assume that *u*=0. Then

$${\lim}_{m \to 0} \frac{s_{q^{*}}\left(m,h,u,Q\right)}{m} = \frac{q^{*}-Q}{q^{*}(1-q^{*})\left(q^{*}+h(1-2 q^{*})\right)} $$

However, we also need to determine when *Δ* *q* has only one root. First we approximate *Δ* *q* by a second-order Taylor series near *q*=0.

$$\begin{aligned} \Delta{q} &\approx s(1-m)\left(1-h(3+2hs)\right) q^{2}\\ &\quad+ \left(hs(1-m)-m\right) q + m Q \end{aligned} $$

Next we find

$$\begin{aligned} &{\lim}_{m \to 0} \frac{\inf\left\{s : \Delta\left(s, m, h, u, Q\right) < 0\right\}}{m} = \frac{1-6Q}{h}\\&+\frac{2 Q-2 \sqrt{Q^{2} + h Q \left(1-3 h (1-3 Q)-6 Q\right) }}{h^{2}} \end{aligned} $$

where *Δ*(*s, m, h, u, Q*) is the discriminant of the Taylor approximation.

Equation 5 is the maximum of these two values.

***Derivation of Eq. 11***

To calculate the change in allele frequency due to selection, when there are many alleles of small effect, we first assume that each copy of a cave-adaptive allele adds $\frac {s}{2 k}$ to the fitness of an individual in the cave population. Therefore, an individual who is homozygous for cave-adaptive alleles at all *k* loci will have a fitness of 1+*s*. Next we assume that the allele frequency of each locus has the same frequency, *q*. Therefore, on average each individual in the population carries 2*k q* copies of cave-adaptive alleles, and the average fitness is $\bar {w} = 1 + (2 k q)(\frac {s}{2 k}) = 1 + q s$. Focusing on a specific locus, and averaging over the other *k*−1 loci, we can calculate marginal genotype fitnesses:

$$\begin{array}{*{20}l} w_{bb} &= 1 + q s \frac{k-1}{k} + \frac{s}{k}\\ w_{Bb} &= 1 + q s \frac{k-1}{k} + \frac{s}{2 k}\\ w_{BB} &= 1 + q s \frac{k-1}{k} \end{array} $$

And the marginal allele fitness for *b* is

$${ \begin{aligned} w_{b} &= q w_{bb} + (1-q) w_{Bb} = 1 + q s \frac{k-1}{k} + q \frac{s}{k}\\ &\quad + (1-q) \frac{s}{2 k}= 1 + q s + (1-q) \frac{s}{2 k} \end{aligned}} $$

Putting this all together:

$$q_{j} = q \frac{w_{b}}{\bar{w}} = q \frac{1 + q s + (1-q) \frac{s}{2 k}}{1 + q s} $$

## Additional file

Additional file 1 Mathematica notebook verifying the analysis of our deterministic model. (NB 115 kb)
